# Measurement of Harm Outcomes in Older Adults after Hospital Discharge: Reliability and Validity

**DOI:** 10.1155/2012/150473

**Published:** 2012-05-09

**Authors:** Alison Douglas, Lori Letts, Kevin Eva, Julie Richardson

**Affiliations:** ^1^School of Rehabilitation Science, IAHS Building, Rm 402, McMaster University, 1400 Main St. W. Hamilton, ON, Canada L8S 1C7; ^2^Department of Rehabilitation Science, McMaster University, Canada; ^3^Centre for Health Education Scholarship, Department of Medicine, University of British Columbia, Canada

## Abstract

*Objectives*. Defining and validating a measure of safety contributes to further validation of clinical measures. The objective was to define and examine the psychometric properties of the outcome “incidents of harm.” *Methods*. The Incident of Harm Caregiver Questionnaire was administered to caregivers of older adults discharged from hospital by telephone. Caregivers completed daily logs for one month and medical charts were examined. *Results*. Test-retest reliability (*n* = 38) was high for the occurrence of an incident of harm (yes/no; kappa = 1.0) and the type of incident (agreement = 100%). Validation against daily logs found no disagreement regarding occurrence or types of incidents. Validation with medical charts found no disagreement regarding incident occurrence and disagreement in half regarding incident type. *Discussion*. The data support the Incident of Harm Caregiver Questionnaire as a reliable and valid estimation of incidents for this sample and are important to researchers as a method to measure safety when validating clinical measures.

## 1. Introduction

Decisions about whether an older adult can live independently require information about safety. Assessment measures currently used by clinicians to aid in determining if a person is safe are typically validated for outcomes that do not include safety. Safety, or the ability to live without unintentional harm or injury, is clinically important in geriatric rehabilitation because of the risk of self-neglect or disorientation, whether or not a person has been diagnosed with frailty or dementia. Concerns arise about unintentional falls, harm from medication errors, or failure to take care of daily needs. Research into safety outcomes is urgently needed to provide information to determine when older adults are at risk of declining health due to self-neglect [1].

Little is known about the ability of assessment tools to predict safety as an outcome. Measures for use with people with dementia have been validated by examining their scores in relation to outcomes such as daily living skills [2–5], discharge status [6, 7], community functioning [8], or likelihood of independent living [3, 5]. These data, although important for establishing the validity of a tool for measuring real-life outcomes, lack a direct link with predicting safety. It is important to establish a reliable and valid outcome measure that addresses safety in the community for older adults.

Initial work has been done to operationalize safety as “incidents of harm” [9]. Incidents of harm were defined as unintentional events that through self-neglect or disorientation (i.e., with no intent of self-harm) lead to: physical injury to self (including exacerbation of chronic illness) or other; and/or damage or loss of property [9]. The intent was to capture outcomes for which persons with cognitive deficits would be more susceptible than persons with intact cognition. In their study, the number of incidents was assessed by administering a telephone questionnaire to the caregiver. Types of incidents were recorded and classified into the following categories: failure to eat and drink; failure to report a medical condition; failure to use prescribed assistive devices; failure to maintain personal hygiene; failure to take medication properly; failure to recognize a familiar environment; failure to turn off electrical appliances; failure to judge fraudulent activities. Results of the study demonstrated that the Mini Mental Status Exam [9] and three neuropsychological tests of recognition memory, executive function, and conceptualization [10] predicted harm in a sample of cognitively impaired people who lived alone. However, data regarding the consistency or accuracy of caregiver reports were not provided. Further validation of the measurement of “incidents of harm” is needed. The accuracy of caregiver report may be challenged, and data demonstrating its accuracy would allow more definitive conclusions regarding the predictive validity of measures for this outcome.

The method of measurement using a telephone interview of a knowledgeable caregiver is a feasible administration method for measuring the outcome “incidents of harm” for several reasons. First, the use of a telephone interview, compared to in-person home visits, requires less time from the study participants, is less burdensome for busy caregivers, and is more cost effective for the research team. Telephone information about incidents is likely to be similar to in-person home visit information because in both cases the research team must rely on reporting of incidents that have occurred in the recent past.

Second, proxy reporting has the advantage of minimizing reporting bias [11] on the part of the older adult, especially if cognitive impairment is suspected. In a population of patients on a geriatric rehabilitation unit such as in this study, some persons may have diagnosis of dementia, whereas others may have physical deficits only, or suspicion of cognitive deficits. Persons with confirmed or possible dementia are likely to have difficulty recalling recent events, may lack insight into abilities, or may have fear of reporting injury. Proxy reporting may minimize reporting bias also by facilitating the person with dementia, who may be suspicious or lack the ability to understand study information, to have comfort with the research study. A caregiver may be more likely to understand explanations about confidentiality and lack of negative consequences in reporting incidents. Cooperation with the research team by a trusted caregiver may be less likely to prompt suspicion on the part of the person with dementia.

Lastly, the use of proxy reporting by a caregiver is supported by literature reviews of patient-proxy reliability, especially when the report is related to observable constructs. Accumulated evidence supports that scores obtained from patients and proxies have good agreement. Patient-proxy agreement is the highest for measures that examine observable behaviours such as eating and dressing [12] or level of functioning [13]. Less observable outcomes such as quality of life or satisfaction showed lower levels of agreement [12, 13].

The objectives of the study were two folds: first, to determine the test-retest reliability of measuring the outcome “incidents of harm” by caregiver interviews and second, to determine the validity of measuring the outcome “incidents of harm” compared to (i) physician reports on medical charts and (ii) caregiver daily logs. The Incident of Harm Caregiver Questionnaire was designed based on the definition of “incidents of harm” from Tierney et al. [9] (see the appendix).

## 2. Methods

### 2.1. Sample

Participants were recruited from consecutive admissions to a geriatric rehabilitation unit. Participants were recruited as part of a broader prospective study, the purpose of which was to examine the predictive validity of predischarge measures to predict postdischarge “incidents of harm” for six months after discharge. The local research ethics board approved the study procedures. Consent was obtained for the research team to access medical records, for the caregiver to be contacted for a telephone interview once per month for six months after the patient was discharged, and for the family physician to be contacted if there was any change in health status. Signed consent from both the patient and caregiver was required. Patient and caregiver pairs were eligible for the study if the patient was over age 65, undergoing assessment for possible dementia (diagnosis of dementia was not required), and the caregiver was identified by the patient and caregiver as the most knowledgeable person about the patient. A diagnosis of dementia was not required in order to broaden the sample, as a portion of inpatients, although suspected of having cognitive deficits and queries about their ability to manage at home, have not been given a formal dementia diagnosis. Exclusion criteria were delirium or a query from the health care team of elder abuse (to avoid possible reporting bias from an abuser).

### 2.2. Procedures

**Figure 1:**
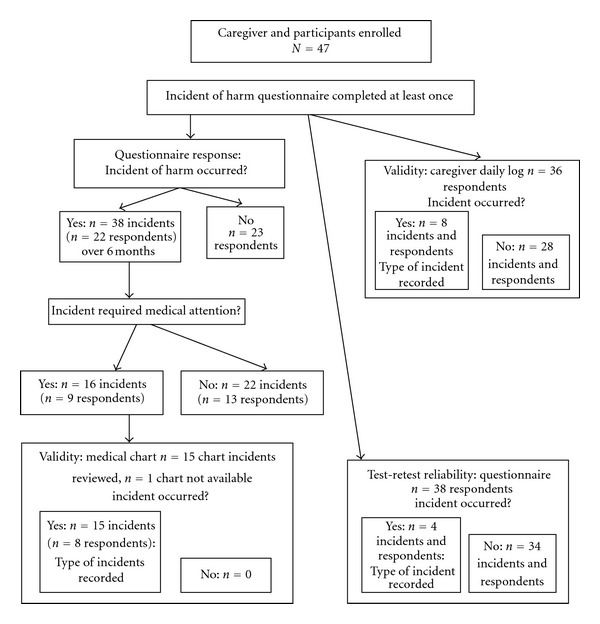
Study procedures.

For this study, the data collection described was completed after patients were discharged from hospital during the six month followup period. A research assistant, who was blind to medical record information, contacted the caregivers by telephone and administered the Incident of Harm Caregiver Questionnaire once each month for six-months (see the appendix for questionnaire). The questionnaire asked the caregiver to identify whether any incident of harm had occurred (yes/no) and whether it was due to self-neglect or disorientation (yes/no, see Figure 1 for study procedures). If an incident was reported, the caregiver was requested to identify from a list the most likely reason for the occurrence (type of incident), or if the caregiver reported being unable to provide a reason or type, the response was recorded as “unknown.” If no incident of harm was noted, the interviewer asked “So is everything going OK?” to gain information about overall functional status and give further opportunity to ask about possible incidents of harm.

For test-retest reliability, caregivers were contacted up to five days after one monthly interview and readministered the questionnaire. The interviewer attempted to contact the caregiver for test-retest administration after the third monthly interview, but if contact was not made within five days, the test-retest administration occurred in a later month. The number and types of incidents of harm were compared between the two interviews. 

For validity, medical record information was reviewed to give a comparison between caregiver and physician report for any incident that required medical intervention. If information about an incident requiring medical attention was not available on the electronic chart, the family physician was contacted by fax to complete a one-page questionnaire [9] (adapted with permission from Dr. Mary Tierney). Caregivers were also requested to keep a daily log of number and types of incidents for either the first or second month after discharge. These were returned by post to the researcher. If the caregiver failed to return the daily log, the researcher phoned to request that it be completed for the current month. New forms were mailed or hand delivered if requested by the caregiver.

### 2.3. Analyses

Test-retest reliability for incident occurrence (yes/no) was calculated using kappa, and for type of incident (failure to complete ADL, etc.) using proportion agreement. Data were collected on the total number of incidents of harm reported by caregivers and the total number of these requiring medical interventions. Only the incidents that required medical intervention were verifiable by checking medical information. The validity of caregiver reported incidents of harm was tested against physician reports and caregiver daily logs using kappa for incident occurrence (yes/no) and proportion agreement for type of incident.

## 3. Results

The sample of patients (*n* = 47) was composed of 55% females with a mean age of 83.3 years including 14 participants over the age of 85 (see Table 1 for patient characteristics). A total of 45 caregivers and patients contributed data to the study and two caregiver participants dropped out by declining to answer or return followup calls. Of the 45 caregiver participants, 39 completed 6 months of followup, 2 died, 2 caregivers dropped out while consenting for their information to remain in the study, and 2 became ineligible due to admission of the older adult to long-term care facilities. Responses were grouped into categories according to the method of Tierney [9] for all incidents (*n* = 35). The highest proportion of incident was of the type “failure to use mobility devices correctly” (*n* = 11, 31.4%), followed by “failure to use medication correctly” (*n* = 7, 20.0%), “failure to perform ADL” (*n* = 6, 17.1%), “failure to report medical condition” (*n* = 6, 17.1%), and “unknown” type (*n* = 5, 14.3%). Test-retest reliability was obtained from 38 caregivers, in the fifth month on average (SD = 1.1), with minimum of two and maximum of five days between test and retest (mean= 3.1, SD = 1.1). The test-retest reliability was high for the occurrence of an incident of harm each month (yes/no, kappa = 1.0) and the type of incident (percent agreement = 100%, Table 2).

 Caregiver reports were validated by checking medical charts or by contacting the family physician regarding any incident that required a medical visit. There were a total of 16 incidents of harm reported by the caregiver that required a medical visit. For a small number of incidents (*n* = 3), information was not available on the hospital chart and verification was obtained by contacting the family physician. A response was received for two of these three incidents and the third incident was noted as a missing data point because of non-response. There was no disagreement with medical charts (kappa = 1.0) (*n* = 15) for the occurrence of an incident of harm (yes/no) (Table 2). 

The type of incident was compared between the caregiver report and notes found on the medical chart (*n* = 15) (Table 3). Any information regarding the possible reason for the medical visit was obtained by reading the entire chart, including physician and other health care team members. The medical chart did not report the type of incident (unknown) in the majority of cases (*n* = 9/15, 60.0%). An example is that the person was seen for an exacerbation of COPD symptoms, and the medical chart did not indicate the type of incident (e.g., failure to take medication correctly). The caregiver stated the incident type was unknown in 1/15 (6.7%). For those incidents for which a type was reported (*n* = 6), there was agreement in 3/6 cases (50%). For those that require medical attention, the most frequent type of incident reported by the caregivers was failure to take medication correctly (*n* = 8/15), and in the majority of these cases (*n* = 6/8, 75%), the medical chart did not report a type of incident. 

The validity of the telephone interview method was also examined by asking caregiver participants to track incidents for one month using a daily log. The return rate for daily logs was 36/47 (76.6%). 20/36 respondents needed more than one reminder to complete the log, or additional mailing of a new copy, having lost the first copy. There was no disagreement between the diary and phone calls about whether the incident occurred (kappa = 1.0, *P* < 0.001) and the types of incidents (percent agreement = 100%). 

## 4. Discussion 

The data support the overall reliability and validity of the measurement of harm outcome. First, the test-retest reliability of the caregiver report by telephone was very high. It was apparent that events of harm that were reported were events that were not easily forgotten in a few days. Nor did recall of the type of event change. There was also high agreement between caregiver report and medical chart data. This indicates that the caregiver report was valid as a measurement of incident of harm outcome using proxy report. In the cases where there was disagreement between the caregiver and the medical chart, the information from the caregiver was more specific about the potential type of incident compared to the medical chart. 

 Although proxy reporters may not have been present for all incidents, such as falls or medication errors, the high agreement with medical chart data demonstrates a lack of bias in caregiver reporting when incidents were serious enough to require medical care. This indicates that any incident noted by the caregiver was reported to the research team. The incident of harm outcome can be viewed as a more objective and observable outcome than, for example, satisfaction, depression, or optimism which have been noted to be less objective and, therefore, more difficult for proxies to rate accurately [12]. The validity of proxy report for the incidents of harm outcome concurs with the literature reporting good patient-proxy agreement with observable behaviours. 

Strong agreement between the telephone interview and daily log further validates the data collection method. It indicates that the method of reporting incidents of harm did not affect whether or not an incident was reported. Although the telephone interview did not specifically ask about the number of incidents, the data from the daily logs showed that some caregivers had noted more than one incident in a month. The telephone interview asked for recall of the entire month, suggesting that it may not have been collecting the same level of detailed data as the diary. Future studies may benefit from modifying the telephone questionnaire to gather data on not only whether an incident occurred in the past month, but also how many occurred. However, the high agreement between daily log and telephone interview for reporting whether an incident occurred validates the telephone interview as an appropriate method for collection of data regarding the occurrence of incidents of harm. Furthermore, the return rate, despite multiple reminders and effort on the part of the researchers, indicated that the daily log was more burdensome for the caregivers, thus making the telephone interview the preferred method of data collection. 

The data from this study indicate that the measurement of incidents of harm through telephone interviews can provide a valid estimate of whether or not an incident occurred according to caregivers and medical staff for this sample. This measurement method is important for validation of clinical assessment tools. It can be used to validate tools for outcomes related directly to safety in the home environment. The outcome “incidents of harm” is a “real-life” outcome that has been noted to be of urgent need in geriatrics [1] and clinical assessment [14]. The outcome “incidents of harm” has value for validation of health care measurement tools used in a broad range of populations and health care settings. The ability to predict community safety is important for clinical assessment with populations such as people with mental illness and acquired brain injury, and the “incidents of harm” outcome quantifies the concept of safety in the real-world setting. The outcome is particularly pertinent in the older adult population for which decisions about independent living and the need for community services often depend on the determination of safety. Future research is needed to examine whether rehabilitation interventions influence this outcome and the characteristics of patients (e.g., age, sex, comorbidities, cognitive status) and caregivers (e.g., live-in or not live-in) who are at greater risk for harm outcome. 

### 4.1. Limitations

Several sources of bias may have influenced the results. The caregivers may have been influenced by reporting and recall bias. Caregivers assume a burden by caregiving, which adds to the stress from various other roles including work and parenting. Caregivers may, therefore, have reported incidents of harm based on their perceived tolerance for risk to the individual. Those who were more anxious about risk may have reported more incidents, those less anxious may have reported an incident but may have stated its consequences were minor and no significant injury occurred. Thus, recall and reporting bias may have either raised or lowered the number of incidents reported. However, when reports by the caregivers were checked against medical charts, the agreement was high, indicating minimal reporting bias for incidents requiring medical intervention. 

Reliability data were based upon reports from telephone interviews of the caregiver, and, therefore, are limited because both sources of data come from the same person. Validity data, although checked against medical records, were also validated using daily logs from the same person, through a different reporting method. Further research is needed to examine whether reliability was influenced by the number of times the measure was administered (i.e., month one versus month 6), and characteristics of the caregiver. 

Proxy report may have lowered the number of incidents reported because proxies may not have been aware of all incidents. However, as previously described, proxy report may have minimized the likelihood of reporting bias on the part of the older adult. The medical chart also may have been influenced by recall and reporting bias such that the medical team may have suspected that the medical visit had been precipitated by disorientation or self-neglect but may not have noted any suspicions on the chart. 

## 5. Conclusions

The telephone interview method for measurement of incidents of harm demonstrated good test-retest reliability and validity in this sample. The measurement of “incidents of harm” by telephone interview with a proxy caregiver showed high agreement with daily logs, while having higher return rates than daily logs. Moreover, it was validated against medical chart reports. A conservative conclusion is that the data from this outcome measurement can be used as a valid estimate of the true occurrence of incidents of harm. More broadly, these data can be used to support the further use of this method of measurement of incidents of harm in research, especially because the outcome “incidents of harm” reflects how a person is managing in the community, for which there is an urgent need. 

## Figures and Tables

**Table 1:** Description of patients.

	Valid (*N*)	Range	Mean	SD
Gender				
Male	21 (44.7%)			
Female	26 (55.3%)			

Age		66–97	83.5	7.7

Years of education		2–17	10.2	3.1

Activities of daily living burden score (FIM)		76−119	103.09	12.25

Co-morbidities index (CIRS-G)		1.30–2.60	2.04	0.27

Cognition score (SMMSE)		10–30	23.79	4.05

FIM: Functional Independence Measure.

CIRS-G: Cumulative Index Rating Scale (Geriatrics).

SMMSE: Standardized Mini Mental Status Exam.

**Table 2:** Test-retest reliability and validity of Incidents of Harm Questionnaire.

	Response (*N*)		*N* with agreement	Valid *N*	Analysis	*P* value
*Test-retest reliability*	Time 1	Time 2				
Incident occurred (yes/no)	yes = 4	yes = 4	38	38	Kappa = 1.0	<0.01
	no = 34	no = 34				
Type of incident			38	38	percent agreement = 100%	

*Validity: medical chart* (only incidents with medical visit, *n* = 15)	Caregiver	Medical chart				
Incident occurred (yes/no)	yes = 15	yes = 15	15	15	Kappa = 1.0	<0.01
	no = 0	no = 0				
Type of incident			3	6	percent agreement = 50%	

*Validity: caregiver daily log*	Phone	Log				
Incident occurred (yes/no)	yes = 8	yes = 8	36	36	Kappa = 1.0	<0.01
	no = 28	no = 28				
Type of incident			36	36	percent agreement = 100%	

**Table 3:** Validity comparison between caregiver and medical chart on type of incident (incidents requiring medical attention only).

Type described by caregiver	Type described by medical chart					
	Failure to do activities of daily living	Failure to take medication correctly	Failure to use mobility devices correctly	Failure to report medical condition	Other	Unknown
Failure to do activities of daily living				1		1

Failure to take medication correctly		1		1		6

Failure to use mobility devices correctly			1			

Failure to report medical condition				1		

Other (substance use)						1

Unknown			1			1

## Appendix

### Incident of Harm Caregiver Questionnaire

(Reference: [9]).

Followup interview checklist: harm outcome

1. *Name of patient participant:*
2. *Outcome: Incidents of harm:* Can you tell me if any of the following have occurred with your—(e.g., parent, spouse) in the past month?
  □(a) any injury to self or another person (including exacerbation of a chronic illness), property loss, or property damage
  □(b) occurrence of the incident due to self-neglect or behaviours related to disorientation (unintentional)
3. In your opinion, what might have been the *most important reason* for the occurrence? (Type of incident)

*ADL: *

Not completing ADL (eating, hygiene, dressing)

Not using medications correctly:

Not using mobility devices correctly:

*IADL: *

Not using kitchen or electrical devices correctly:

Not detecting fraud:

Not reporting a medical condition:

*Environment: *

Not recognizing a familiar environment:

Unsafe home set-up:

Other:

(4) Note whether medical attention was sought and from where (e.g., hospital, family physician)
(5) If no incident of harm is reported, ask “So, is everything going OK?”
